# Arthroscopic intralesional curettage for large benign talar dome cysts

**DOI:** 10.1051/sicotj/2015032

**Published:** 2015-12-01

**Authors:** Ossama El Shazly, Maged M. Abou El Soud, Nasef Mohamed Nasef Abdelatif

**Affiliations:** 1 Orthopedic Department of Ain Shams University 11517 Cairo Egypt; 2 Faculty of Medicine, Bani Suef University Teaching Hospitals 62511 Bani Suef Egypt

**Keywords:** Hindfoot endoscopy, Talar dome cysts, Aneurysmal bone cyst talus

## Abstract

*Introduction*: Surgical management of large talar dome cysts is challenging due to increased morbidity by associated cartilage damage and malleolar osteotomy. The purpose of this study is to evaluate the clinical and radiological outcome of endoscopic curettage and bone graft for large talar dome cysts.

*Methods*: This is a retrospective analysis of data for eight patients (eight feet) who were treated by arthroscopic curettage and grafting for large talar dome cysts. Seven cases were treated by posterior ankle arthroscopy as the lesion was located posteriorly while one case was treated by anterior ankle arthroscopy as the lesion was breached anteriorly.

*Results*: The final diagnosis, was; large osteochondral lesion of talus (two cases), aneurysmal bone cyst (ABC) (two case), intra-osseous ganglion (two cases), Chronic infection in talus (one case) and angiomatous lesion of the talus (one case). The mean follow up period was 18.3 (±3.06 *SD*) months (range 16–25 months). The median preoperative AOFAS score was 74.5 (±5.34 *SD*) points. The mean postoperative AOFAS score at one year follow up was 94.6 (±2.97 *SD*) points. None of the patient had recurrence of the lesion during follow up. Return to normal daily activity was achieved at 11.25 (±2.37 *SD*) weeks.

*Discussion*: In this short case series study, large talar dome bony cysts of different pathologies including aneurysmal bone cysts could be treated effectively by endoscopic curettage and bone grafting with no recurrence no complications during the follow-up period.

## Introduction

Large bony cysts in the talar dome are encountered in different pathological conditions such as aneurysmal bone cysts (ABCs), intra-osseous ganglia, simple bone cysts, and large osteochondral lesions of the talus [1]. Most of the reports suggest intralesional curettage and bone grafting for localized lesions. In certain conditions when the lesion is large with considerable talar dome damage, fresh osteochondral allografts or vascularized bone grafts can be used. Talectomy followed by tibiocalcaneal arthrodesis is indicated when failure of salvage procedures of the talus is encountered [2–5]. Open surgical approach requires medial malleolar osteotomy in order to access these lesions. In most instances, the cartilaginous roof of the cyst is scarified in order to perform proper curettage and bone grafting. Few reports on arthroscopic management of these cysts have been published [6–10]. “Intralesional arthroscopy” can provide good visualization for these lesions without osteotomy of the medial malleolus or jeopardizing the articular cartilage. This study is a retrospective case series study on eight patients who had large talar dome cysts of different aetiologies. The purpose of this study is to evaluate the clinical and radiological outcome of arthroscopic curettage and bone grafting for large talar dome cysts. We believe that the arthroscopic procedure can effectively treat these lesions.

## Patients and methods

**Figure 1.**
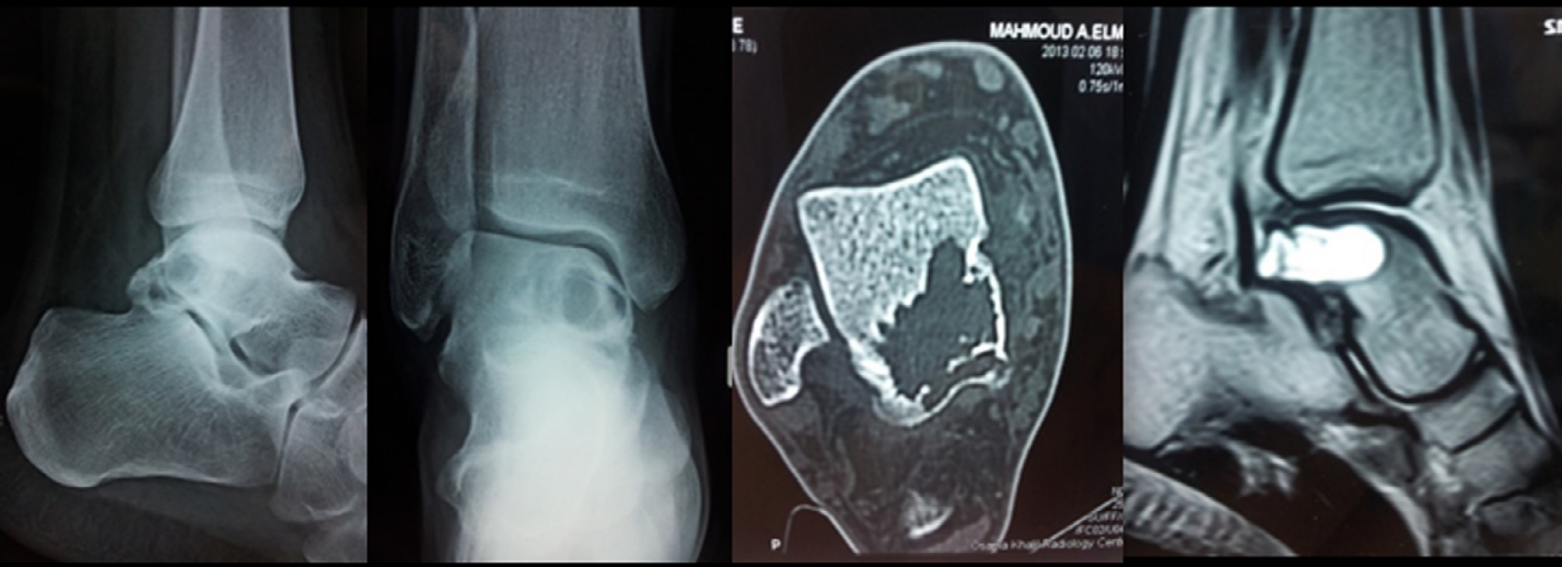
A male patient 28 years old. Plain radiographs, CT scan, and MRI revealed a large aneurysmal bone cyst involving the posterior third of talus.

This is a retrospective analysis of data for eight patients (eight feet) who were treated by arthroscopic curettage and bone grafting for large talar dome cysts. All patients complained of deep ankle pain which increased with activity. Routine radiographs, CT scans and MRI were performed preoperatively done for all cases. The radiological reports confirmed the presence of a large benign cystic osteolytic lesion in the talar dome in all cases. The decision for arthroscopic intralesional curettage was only done when the cyst diameter was 10 mm or more in the preoperative CT scan. Smaller diameter cysts would not accommodate the arthroscopic tools. Centrally located cysts were not treated by this procedure as they were difficult to approach whereas posterior or anteriorly localized cysts were easily approached via posterior or anterior arthroscopy, respectively (Figure 1).

The preoperative provisional diagnosis, based on radiological findings, was: large osteochondral lesion of talus (four cases), aneurysmal bone cyst (ABC) (two cases), intra-osseous ganglion (two cases) (Table 1).

**Table 1.** The difference between preoperative and postoperative diagnosis.

	Preoperative diagnosis	Histopathological findings	Final diagnosis
1.	Osteochondral bone cyst	Necrotic subchondral bone trabeculae	Osteochondral bone cyst
2.	Osteochondral bone cyst	Necrotic bone with infilteration by plasma cells and scattered lymphocytes and polymorpholeucocytes	Chronic infection in talus
3.	Aneurysmal bone cyst	Eroded bone with numerous multinoculated giant cell with inflammatory cells amidst pools of blood	Aneurysmal bone cyst
4.	Intraosseus ganglion	Fibrous tissue with mucoid degeneration	Intraosseus ganglion
5.	Osteochondral bone cyst	Necrotic subchondral bone trabeculae	Osteochondral bone cyst
6.	Aneurysmal bone cyst	Eroded bone with numerous multinoculated giant cell with inflammatory cells amidst pools of blood	Aneurysmal bone cyst
7.	Osteochondral bone cyst	Sclerotic bone surrounding vascular tissue with endothelium lining	Angiomatous lesion of the talus
8.	Intraosseus ganglion	Fibrous tissue with mucoid degeneration	Intraosseus ganglion

All cases signed a preoperative informed consent describing the procedure as well as all the possible complications.

Seven cases were treated by posterior ankle arthroscopy as the lesion was located posteriorly while one case was treated by anterior ankle arthroscopy as the lesion was breached anteriorly. The procedure involved arthroscopic curettage and bone grafting of the lesion. Cancellous iliac autograft was used in seven cases, while artificial bone granules were used in one case. Intralesional biopsy was taken during the procedure from all lesions to confirm the diagnosis.

### Technique

#### Posterior ankle arthroscopy

**Figure 2.**
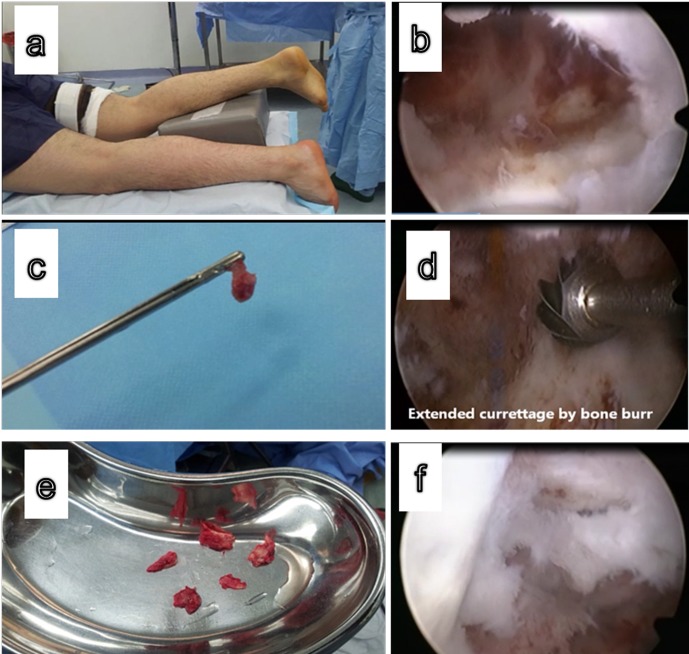
(a) Prone position of the patients with operated leg on sand bag, (b) arthroscopic view of the orifice of the cyst posteriorly, (c) sampling of the membrane of cyst of histopathological examination, (d) extended curettage of the cyst by bone burr, (e) iliac bone graft, (f) packing of the cyst by bone graft.

Four patients received general anesthesia while three patients received spinal anesthesia. The patient was placed in the prone position with the affected ankle placed over a sand bag. The two portal technique described by Van Dijk was used [6]. Through the lateral portal, visualization of the lesion was done and identification of the flexor hallucis (FHL) tendon was confirmed by mobilization of the big toe. The FHL tendon was an important landmark for the proximity of the neurovascular bundle. During the whole procedure, attention was directed to not crossing medially to the FHL to avoid injury of the neurovascular bundle. The removal of a bone window from the posterior process of the talus was done to visualize the cyst from inside. This was done easily by removal of the thin posterior wall of the cyst using basket forceps till the cyst was visualized from inside. The scope was introduced inside the cyst, and then a biopsy was taken from the lesional membrane. Curettage was done using bone curettes, shavers, and bone burrs. Characteristically, in aneurysmal bone cyst “welling” of blood was seen once the membrane of the cyst was touched by the instruments, confirming the diagnosis of ABC. In this case, an additional extended curettage was done using high-speed burr. Also phenol 5% was infiltrated inside the lesion as an adjuvant therapy. Corticocancellous iliac bone graft was taken from the ipsilateral iliac bone and the graft pieces were packed inside the lesion under endoscopic guidance (Figures 2 and 3 – Video).

**Figure 3.**
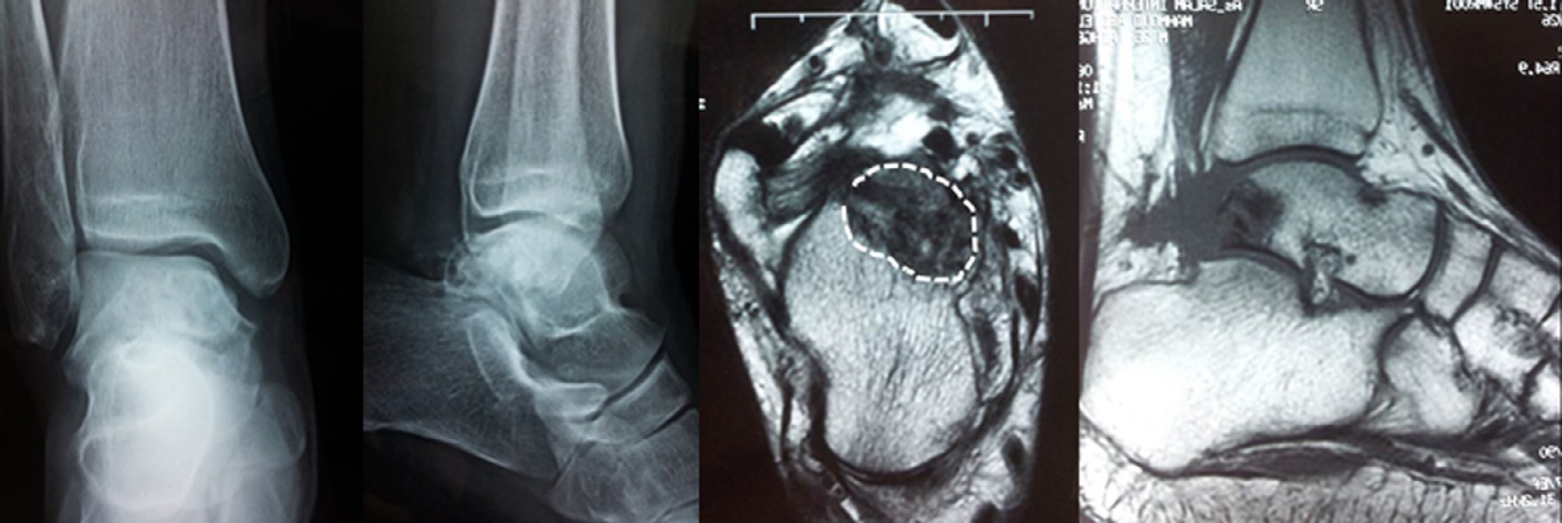
Radiological evaluation of the cyst after 2 years by plain X ray and MRI showing complete healing of the cyst with no recurrence.

#### Anterior ankle arthroscopy

**Figure 4.**
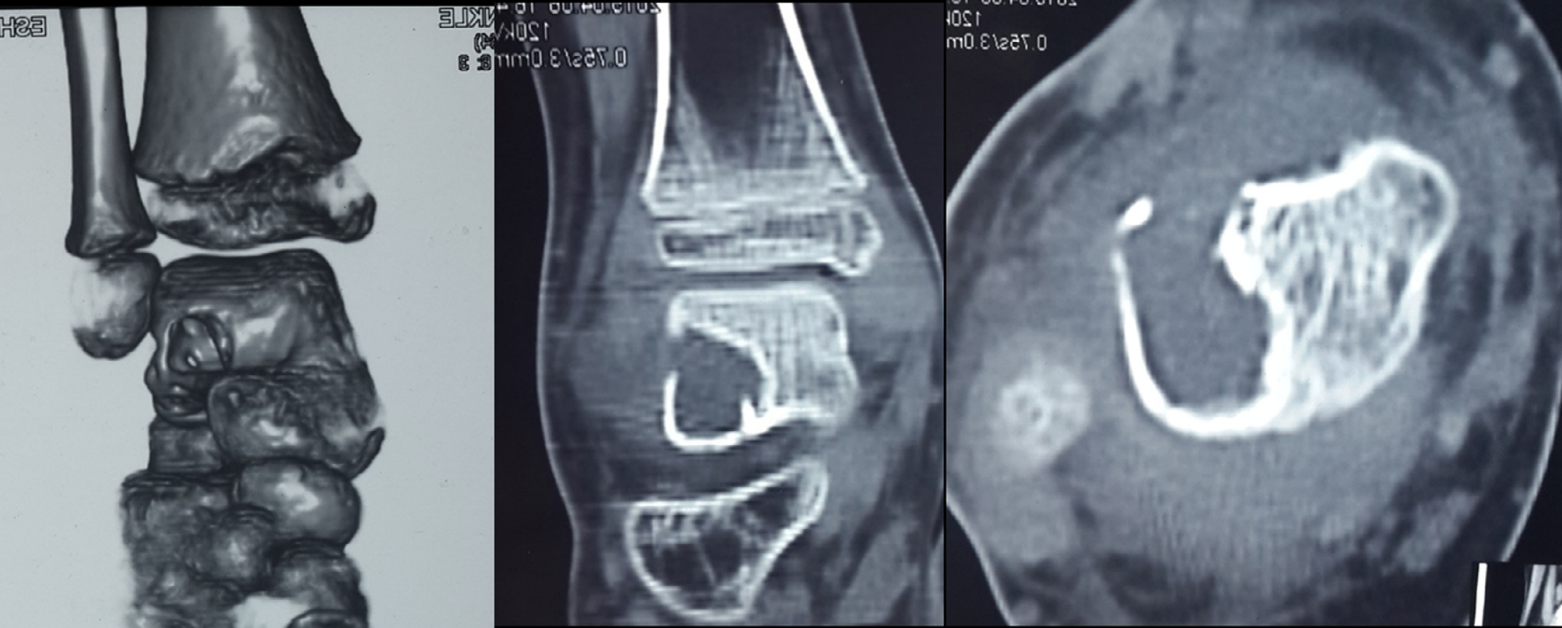
CT scan of an 8 year-old child showing a large aneurysmal bone cyst eroding the anterolateral cortex of talus.

**Figure 5.**
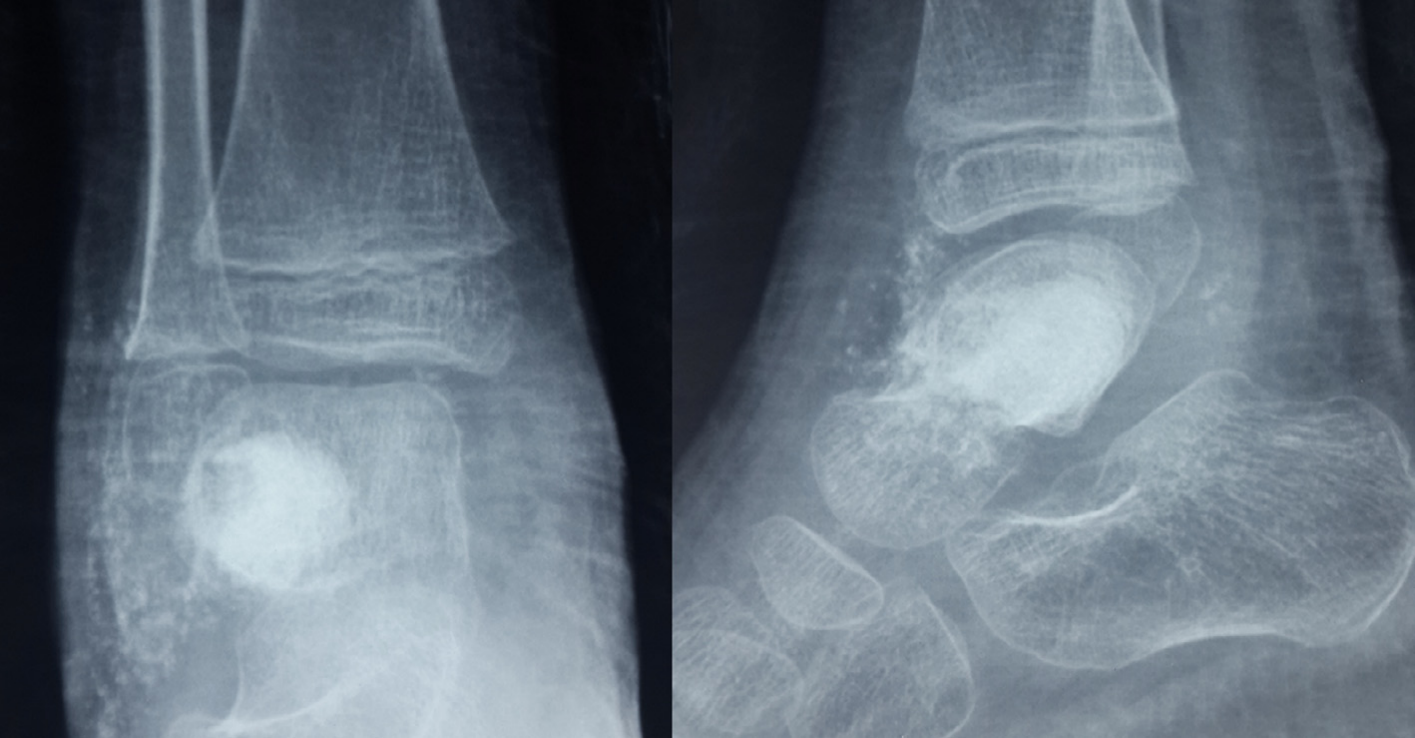
Sixteen months of follow-up after curettage of the cyst and impaction by bone granules.

One case was treated by anterior ankle arthroscopy. The patient received general anesthesia and he was positioned in a supine position with the operated foot located off the edge of the table. In this case, the lesion was a large aneurysmal bone cyst, which was located anteriorly causing a small erosion in the anterolateral cortex of the talus. Widening of the hole was done with basket forceps. Then curettage and bone biopsy was done. In this case artificial bone granules were used instead of bone graft because the patient was skeletally immature (Figures 4 and 5).

### Postoperative management

Immobilization was for 4–6 weeks in a long leg cast. Partial weight bearing was allowed after six weeks.

Postoperative evaluation was done via clinical and radiological assessment.

Functional evaluation used the AOFAS score for the ankle [11].

## Results

The study was conducted on eight patients (eight feet), six males and two females. The mean age of the patients was 23.6 (±8.33 *SD*) years, range (8–36 years). The mean follow-up period was 18.3 (±3.06 *SD*) months (range 16–25 months). The median preoperative AOFAS score was 74.5 (±5.34 *SD*). The mean postoperative AOFAS score at one year follow-up was 94.6 (±2.97 *SD*). None of the patients had recurrence of the lesion during follow-up. Return to normal daily activity was achieved at 11.25 (±2.37 *SD*) weeks. Histopathological examination confirmed the radiological diagnosis in six cases, while the diagnosis was changed in two cases (Table 1).

## Discussion

Large talar dome cysts are formed due to different aetiologies. These cysts are usually painful due to increased intra-osseous pressure and most of the patients have repeated ankle swelling with activity [12]. Whatever the aetiology, debridement and bone grafting is the gold standard treatment [3]. However when a neoplastic origin is suspected, extended curettage may be needed with adjuvant therapy such as infiltration of phenol 5% or liquid nitrogen [1]. Open surgical management of these lesions is associated with extensive soft tissue dissection, damage of articular cartilage, medial malleolar osteotomy, and increased morbidity. Very few reports have been published on arthroscopic management of these lesions. The arthroscopic technique was performed as described by Van Dijk [6, 7].

Lui [8, 9] reported a case of arthroscopic management of large osteochondral lesion of cyst by curettage and bone grafting. He reported improvement of patients’ symptoms and absence of recurrence after 28 months of follow-up. Ogut et al. [10] reported the outcome of six ankles (five patients) with posteriorly localized talar cysts treated by endoscopic debridement and curettage. The sole pathology in their series was an osteochondral lesion of talus. All patients reported excellent results by AOFAS score after one year follow-up. In our study, the underlying pathology was variable. Two cases had aneurysmal bone cysts of talus which is rarely encountered. Sharma et al. [1] stated that less than 20 cases were reported on PubMed till 2012. In our study, one case was adult and the other was a young child. The recommended treatment for ABC is intralesional extended curettage with adjuvant therapy, and then filling of the lesion with bone graft or cement [1, 13]. This treatment could be applied arthroscopically in our series. Extended curettage was done using a high-speed bone burr, and adjuvant therapy was applied by infiltration of phenol 5% to kill the tumour cells. Grafting is recommended in large cystic lesions of the talus in order to provide a mechanical support to the articular surface. We used corticocancellous grafts in all cases except in one case in whom bone substitute was used due to skeletal immaturity of the patient. Preoperative diagnosis was changed postoperatively after histopathological examination in two cases (case number 2 and case number 7). These two cases had a preoperative diagnosis of osteochondral lesion of talus, which was changed to chronic nonspecific infection in case number 2 and angiomatous lesion of the talar dome in case number 7 (Table 1). Although the change of diagnosis had no impact on the surgical management, postoperatively the case of chronic nonspecific infection required treatment with broad spectrum antibiotic for four weeks. None of the patients in our series had recurrence during the period of follow-up, and no complications related to the procedure were reported. One of the limitations of our study was the small number of cases, and the marked variability in the diagnosis which made the statistical significance of our results questionable. However, this was attributed to the paucity of such cases in practice. Based on PubMed search this study is considered at this time the largest case series study of endoscopic management of large talar dome cysts.

## Conclusion

In this short case series study, large talar dome bony cysts of different pathologies including aneurysmal bone cysts could be treated effectively by arthroscopic curettage and bone grafting with no recurrence or complications during the follow-up period.

## Conflict of interest

The authors declare no conflict of interest in relation with this paper.

## Supplementary material

**Video 1** – Arthroscopic currettage. **Video 2** – Bone grafting for ABC lesion of talus.
